# Fatal outcome after self-expanding transcatheter aortic valve replacement of the bicuspid valve due to infolding: a case report

**DOI:** 10.1186/s44215-025-00224-3

**Published:** 2025-10-14

**Authors:** Nagi Hayashi, Junji Yunoki, Keijiro Shigetomi, Kouhei Baba, Masahide Shichijo, Koki Jinnouchi, Hiroyuki Morokuma, Manabu Itoh, Keiji Kamohara

**Affiliations:** Department of Thoracic and Cardiovascular Surgery, Saga University, 5-1-1 Nabeshima, Saga, Saga 849-8501 Japan

**Keywords:** TAVR, Bicuspid valve, Infolding, Self-expandable valve

## Abstract

**Background:**

Infolding is a rare but potentially life-threatening complication of self-expanding transcatheter aortic valve replacement (TAVR).

**Case presentation:**

We describe the case of an 80-year-old man who was referred for the treatment of severe aortic stenosis complicated by heart failure. Transthoracic echocardiography revealed the following: peak velocity, 6.1 m/s; mean pressure gradient, 102 mmHg; and aortic valve area, 0.26 cm^2^. Computed tomography (CT) showed a bicuspid aortic valve with a R-L raphe, an annular area of 529 mm^2^, and a perimeter of 83.4 mm. Considering the patient’s severe emphysema, transfemoral TAVR was performed with the patient under deep sedation. A 34-mm Evolut FX valve was implanted after predilation with a 20-mm Inoue balloon. During deployment up to the point of no recapture, hypotension occurred without improvement, and mild infolding was suspected. However, full deployment was performed, as valve optimization was considered likely to stabilize hemodynamics. Post-deployment balloon dilation was performed; however, valve under-expansion and moderate aortic regurgitation persisted. Initial hemodynamics were stabilized; however, the patient gradually developed respiratory distress. Follow-up CT confirmed substantial valve infolding. Pulmonary hypertension, alveolar hemorrhage, and disseminated intravascular coagulation developed. Surgical aortic valve replacement with a 21-mm valve was performed 15 days post-TAVR. The explanted TAVR valve exhibited marked structural deformation. Although the patient’s circulatory status improved postoperatively, he died of respiratory failure due to pneumonia.

**Conclusion:**

This case highlights the importance of comprehensive preoperative anatomical assessment and intraoperative decision making in high-risk patients, particularly those with bicuspid valves. Valve selection (considering the valve type and size) must be meticulously tailored to the anatomical features surrounding the annulus. In addition, upon its recognition, substantial infolding should be promptly addressed by recapturing the valve, adjusting the valve size, or redeploying the valve with additional balloon aortic valvuloplasty.

## Background

Infolding is a rare complication associated with self-expanding valves that can occasionally result in a fatal outcome. It is more likely to occur in cases involving bicuspid valves or the use of large implants. Here, we describe a case of infolding that led to a fatal outcome after transcatheter aortic valve replacement (TAVR) of the bicuspid valve.

### Case presentation

#### Patient information and clinical findings

The patient was an 80-year-old man who was a current smoker. A systolic murmur was auscultated at the second right sternal border. The patient was classified as having New York Heart Association Class III heart failure. The Clinical Frailty Scale score was 3. The calculated risk scores were as follows: EURO II score, 1.9%; Society of Thoracic Surgeons, 3.8%; and Japan score, 6.6%. Blood test results showed a hemoglobin level of 12.2 g/dL, indicating the absence of anemia. Additionally, no hepatic or renal dysfunction was observed. The N-terminal pro-B-type natriuretic peptide level was mildly elevated at 2406 pg/mL. Electrocardiography showed normal sinus rhythm, and no bundle branch block was detected. Pulmonary function test results showed a percent vital capacity of 92.4% and percent forced expiratory volume in 1 s of 48.1%, indicating obstructive pulmonary impairment.

Echocardiography revealed an ejection fraction (EF) of 66%, satisfactory wall motion, aortic valve peak velocity of 6.1 m/s, mean pressure gradient (PG) of 102 mmHg, and aortic valve area (AVA) of 0.27 cm^2^. Mild aortic regurgitation (AR) and mitral regurgitation were observed, and the valve was bicuspid.

**Fig. 1:**
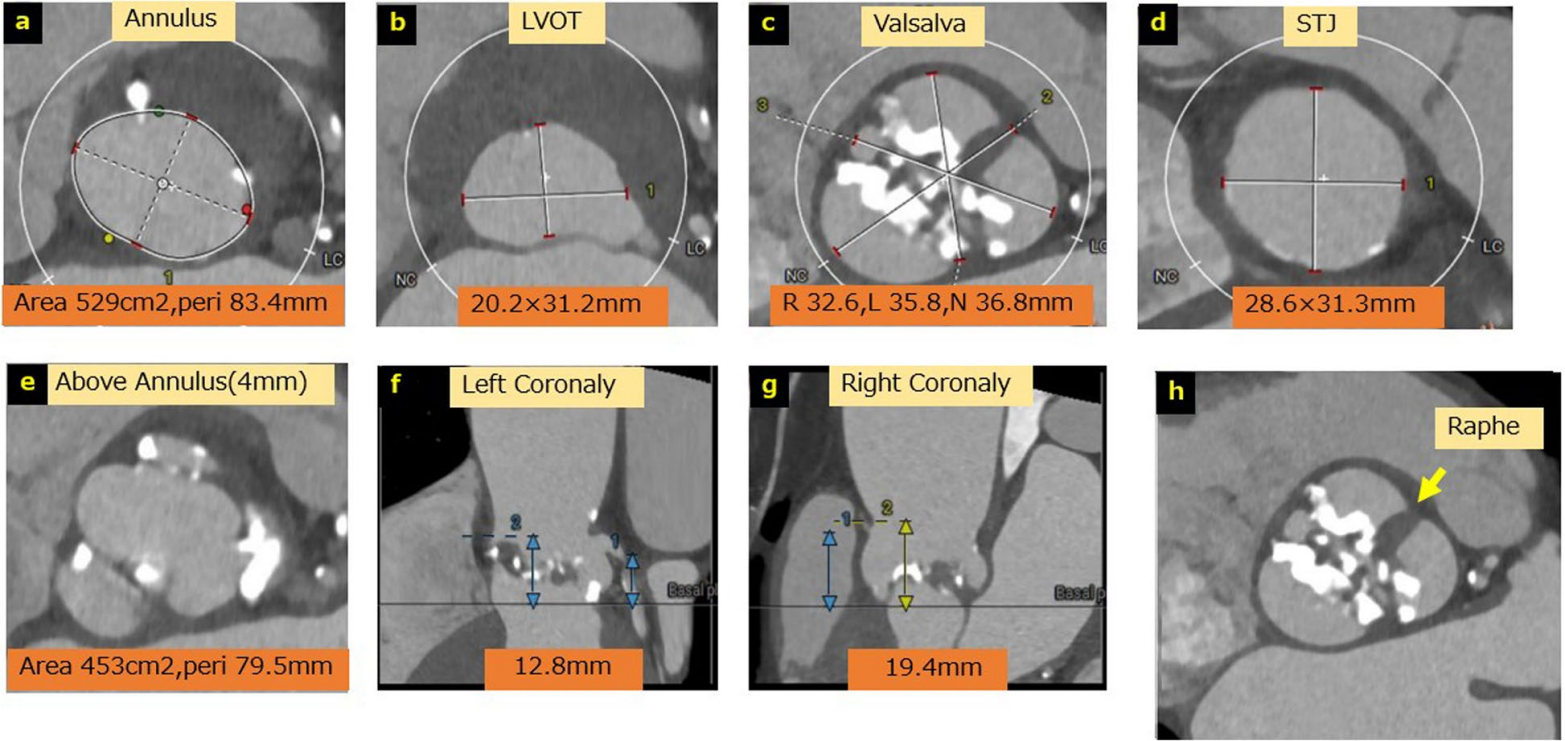
Preoperative contrast-enhanced computed tomography image of transcatheter aortic valve replacement. **a** Annulus; **b** left ventricular outflow tract; **c** Valsalva sinus; **d** sinotubular junction; **e** area 4 mm above the annulus; **f** left coronary height; **g** right coronary height; h: bicuspid valve, a type 1 bicuspid aortic valve with a R-L raphe is identified. *LVOT* left ventricular outflow tract, *STJ* sinotubular junction

Contrast-enhanced computed tomography (CT) revealed a minimal iliac artery diameter of 5.8 mm, suggesting that transfemoral access would be barely feasible. The aortic annulus measured 529 mm^2^ (21.3 × 31.1 mm; mean diameter, 26.2 mm) with a perimeter of 83.4 mm. The Valsalva sinus measured 32–36 mm, and the sinotubular junction measured 28 × 31 mm. The height of both coronary ostia exceeded 12 mm. No coronary artery stenosis was observed; however, the patient had a Sievers classification type 1 bicuspid aortic valve (BAV) with a raphe between the left and right coronary cusps [1]. The cross-sectional area 4 mm above the annulus was 453 mm^2^ (Fig. 1a–h).

#### Treatment strategy

Although the patient was considered low-risk, advanced age and impaired respiratory function led to the decision that performing TAVR with the patient under local anesthesia with sedation was preferable. A 34-mm Evolut FX valve (Medtronic, Minneapolis, MN, USA) was used.

#### Operative findings

**Fig. 2:**
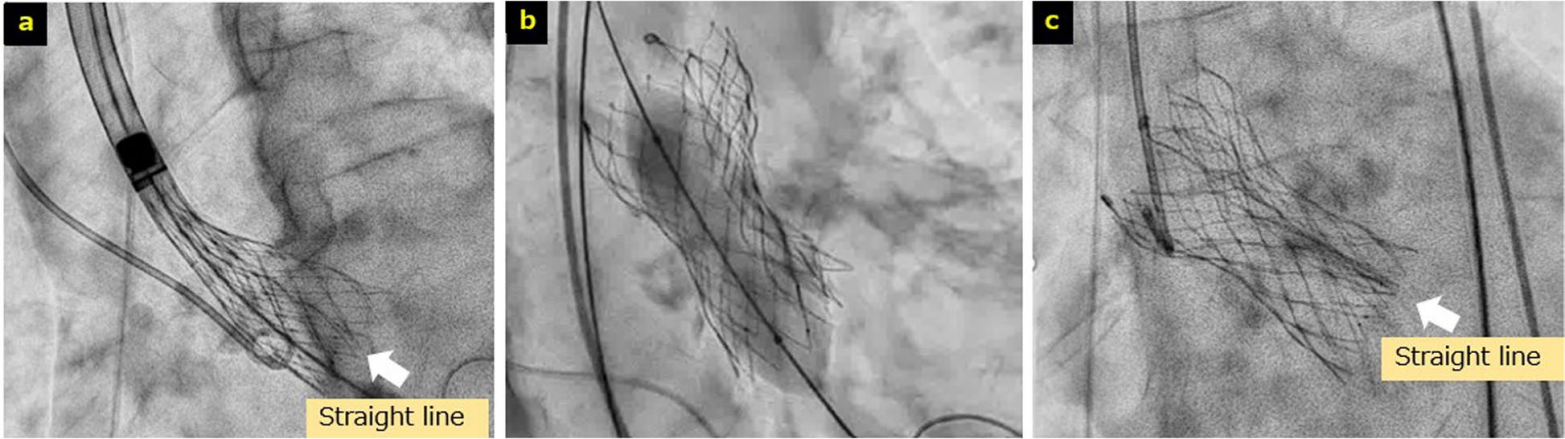
Intraoperative angiography during transcatheter aortic valve replacement. **a** The point of no recapture. A straight line (white arrow) is observed suggestive of valve infolding. **b** Post-balloon aortic valvuloplasty. **c** Infolding persists even after balloon aortic valvuloplasty

Local anesthesia with deep sedation was administered, and vascular access was obtained via the right femoral artery using the Perclose ProStyle System (Abbott, Santa Clara, CA, USA). Following predilation with a 20-mm Inoue balloon (Abbott, Santa Clara, CA, USA), the 34-mm Evolut FX valve was advanced across the aortic valve. Immediately after crossing the valve, hypotension was observed, raising the suspicion of AR and prompting rapid valve deployment. Despite deployment up to the point of no recapture (PNR), the patient’s blood pressure response remained suboptimal, and persistent AR was suspected. Although findings suggestive of valve infolding were noted, we hypothesized that full expansion might improve the patient’s hemodynamics; therefore, valve deployment was continued (Fig. 2a).

Post-deployment, the patient’s blood pressure stabilized; however, valve infolding and moderate AR were confirmed. Consequently, post-dilation was performed using a 22-mm TRIVAL balloon (Kaneka Medix Corporation, Osaka, Japan) (Fig. 2b). Although valve recoil was observed, the degree of AR was reduced, and the procedure was completed (Fig. 2c).

**Fig. 3:**
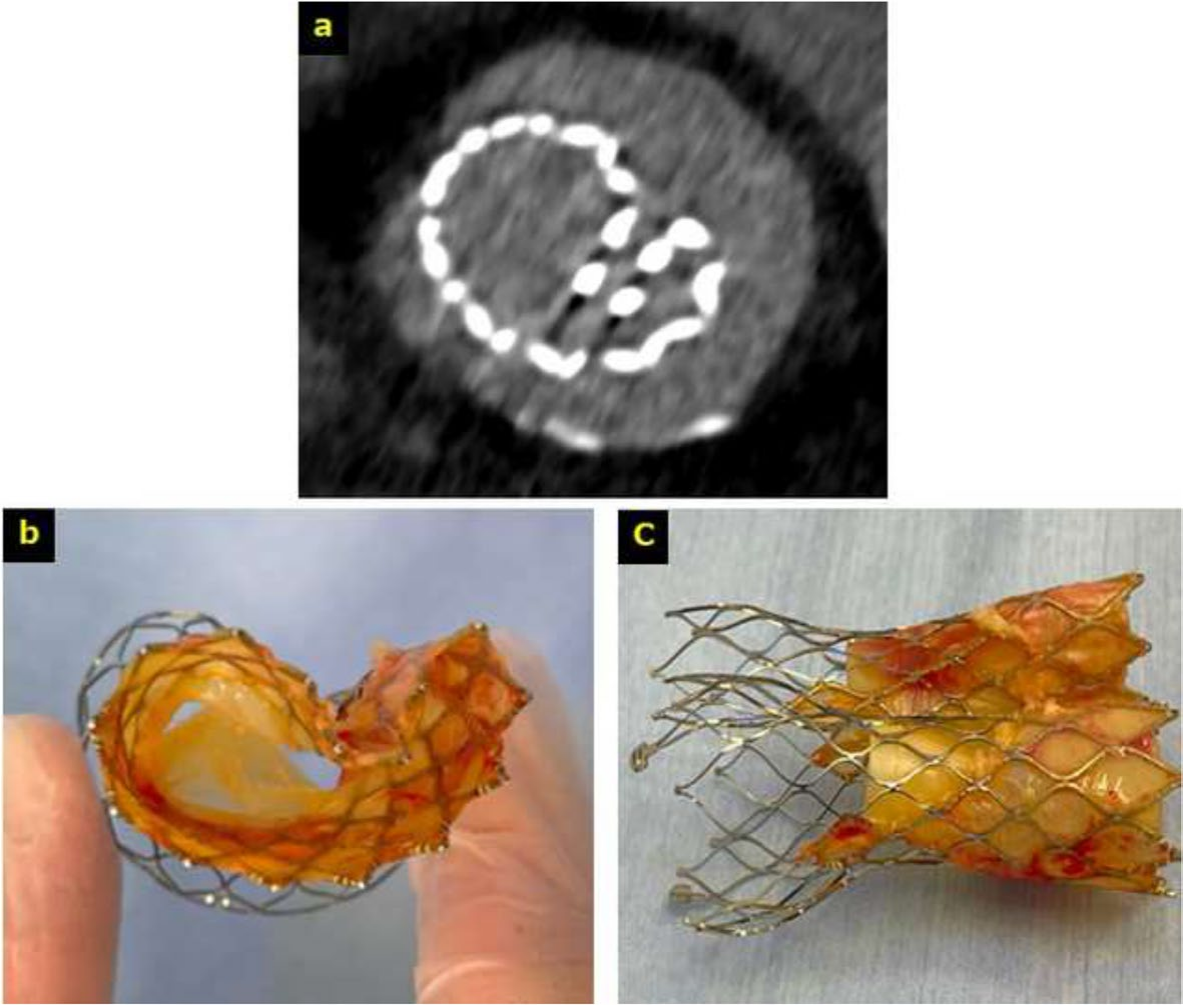
Imaging of infolding and explantation. **a** Postoperative computed tomography image showing that the valve is severely infolded. **b**, **c** The explanted Evolut FX valve

Dobutamine was discontinued on the day following surgery. No atrioventricular block was observed, and the patient was transferred from the intensive care unit on postoperative day (POD) 2. Although rehabilitation was initiated thereafter, the patient developed dyspnea as it progressed. Postoperative echocardiography revealed an EF of 64%, moderate to severe AR, and the following aortic valve measurements: peak velocity, 3.0 m/s; mean PG, 19 mmHg; and AVA, 0.70 cm^2^. CT revealed marked, heart-shaped, and severe infolding (Fig. 3a).

Subsequently, the patient developed hemoptysis, necessitating reinitiation of dobutamine. An alveolar hemorrhage was identified, prompting endotracheal intubation and steroid pulse therapy. Disseminated intravascular coagulation secondary to the hemorrhage was also observed. Once the patient’s condition stabilized with treatment, surgical aortic valve replacement was performed. Renal dysfunction and a transient atrioventricular block were noted preoperatively. On POD 15, reoperation via median sternotomy was performed. The TAVR valve was explanted, and surgical aortic valve replacement was performed using a 21-mm Avalus valve (Medtronic) (Fig. 3b, c). Although hemodynamic stability was achieved postoperatively, the patient’s respiratory status remained poor and was complicated by infection. Ultimately, the patient died of respiratory failure due to pneumonia on POD 11 after reoperation.

## Discussion and conclusions

The infolding of self-expanding valves is a rare but clinically important complication that can precipitate acute AR and profoundly compromise a patient’s hemodynamic stability. Although reported in approximately 1–3% of TAVR cases, it remains underrecognized [2, 3]. This phenomenon has been associated with adverse outcomes, e.g., renal dysfunction and complete atrioventricular block, and in some cases, it results in cardiac arrest, underscoring the need for heightened clinical vigilance [4, 5]. Given the increasing number of TAVRs performed worldwide, awareness of infolding and its appropriate management is of critical importance. This case underscores several key lessons regarding risk assessment, valve sizing, balloon selection, and intraoperative decision making.

First, it is essential to recognize that our patient had several known anatomical and procedural risk factors for infolding. Some studies have reported anatomical risk factors, e.g., high ellipticity of the annulus, eccentric or heavy calcification, and bicuspid native valve, and device-related risk factors, e.g., larger prosthetic valve, re-sheathing, and improper loading [3, 4, 6]. Particularly, a combination of them make patients at high risk for incomplete prosthesis expansion, malapposition, and infolding. In such cases, extremely cautious pre-procedural planning is vital.

One critical issue in this case was the selection of a 34-mm valve. The measured annular area and perimeter placed the anatomy at the borderline between 29-mm and 34-mm valve sizes. Importantly, the cross-sectional area measured 4 mm above the annulus was only 453 mm^2^, strongly favoring the use of a 29-mm valve [7, 8]. In hindsight, choosing a 34-mm prosthesis likely represented an oversizing error, increasing the risk of infolding. The decision-making process for selecting the larger valve should have been more critically evaluated pre-procedurally.

Second, the choice of balloon for pre-dilation warrants reconsideration. A 20-mm Inoue balloon, which is soft, was used for pre-dilatation before deploying a 34-mm Evolut valve. Strong balloon pre-dilatation and post-dilatation may increase the risk of annular rupture in heavily calcified BAVs [9]. At our institution, we have previously encountered cases of annular rupture during pre-dilatation in patients with BAVs, which influenced the decision to use a smaller, more compliant balloon in the present patient. However, this conservative approach may have contributed to the development of valve infolding. While care must be taken to avoid annular rupture, effective pre-dilatation in patients with severely calcified BAVs—particularly those with rigid leaflets or raphe—often necessitates the use of a larger balloon diameter or one with greater radial force to achieve adequate expansion and facilitate optimal valve deployment.

Third, the issue was how to manage intraoperative infolding when it was identified. When infolding is suspected, confirmation using multiple fluoroscopic views is recommended for accurate assessment [6]. In the current case, vertical lines and suboptimal expansions were observed at the PNR. Although hypotension suggestive of AR was noted, it was determined that complete expansion would maintain hemodynamic stability better than incomplete expansion. Therefore, full expansion was pursued; however, substantial infolding persisted. In minor infolding cases, post-dilatation leads to improvement, whereas in other cases, valve replacement with a newly downsized prosthesis or transcatheter aortic valve (TAV)-in-TAV implantation may be required [2, 3, 10]. Hence, when infolding is diagnosed promptly at the PNR, prosthesis removal and replacement is the recommended approach, rather than post-dilatation [10]. In the present case, recoil was observed and sufficient expansion could not be attained. Some patients experience cardiac arrest immediately after deployment and may require mechanical circulatory support [11]. Appropriate intervention before full expansion is crucial to prevent severe outcomes, and rather than relying on post-dilatation, prompt valve replacement should be performed without hesitation if infolding is identified at the PNR.

Postoperatively, the patient’s symptoms progressively worsened with the advancement of rehabilitation. Given the pre-existing severe folding, it is conceivable that the stiffening and expansion of the prosthetic valve led to the exacerbation of AR.

To prevent severe outcomes associated with infolding, a thorough preoperative risk assessment, vigilant intraoperative observation, and accurate judgment before postoperative deterioration are essential. This case serves as a didactic example illustrating that a fatal outcome could likely have been avoided at multiple critical junctures, and it might have been avoidable if an appropriate and timely decision had been made.

This case highlights the importance of comprehensive preoperative anatomical assessment and intraoperative decision making in high-risk patients, particularly in those with bicuspid valves. Valve selection must be meticulously tailored to the anatomical features surrounding the annulus. In addition, upon its recognition, substantial infolding should be promptly addressed by recapturing the valve, adjusting the valve size, or redeploying the valve with additional balloon aortic valvuloplasty. In cases where a valve with infolding has been deployed, the surgical intervention should not be delayed.

## Data Availability

All data generated or analyzed during this study are included in this published article.

## Abbreviations

TAVR: Transcatheter aortic valve replacement
EF: Ejection fraction
PG: Pressure gradient
AVA: Aortic valve area
AR: Aortic regurgitation
CT: Computed tomography
POD: Postoperative days
TAV: Transcatheter aortic valve
BAV: Bicuspid aortic valve
PVL: Paravalvular leak
PNR: Point of no recapture
